# Virulence gene profiles and phylogeny of Shiga toxin-positive *Escherichia coli* strains isolated from FDA regulated foods during 2010-2017

**DOI:** 10.1371/journal.pone.0214620

**Published:** 2019-04-01

**Authors:** Narjol González-Escalona, Julie Ann Kase

**Affiliations:** Division of Microbiology, Center for Food Safety and Applied Nutrition, Food and Drug Administration, College Park, MD, United States of America; USDA-ARS Salinity Laboratory, UNITED STATES

## Abstract

Illnesses caused by Shiga toxin-producing *Escherichia* coli (STECs) can be life threatening, such as hemolytic uremic syndrome (HUS). The STECs most frequently identified by USDA’s Microbiological Data Program (MDP) carried toxin gene subtypes *stx1a* and/or *stx2a*. Here we described the genome sequences of 331 STECs isolated from foods regulated by the FDA 2010–2017, and determined their genomic identity, serotype, sequence type, virulence potential, and prevalence of antimicrobial resistance. Isolates were selected from the MDP archive, routine food testing by FDA field labs (ORA), and food testing by a contract company. Only 276 (83%) strains were confirmed as STECs by *in silico* analysis. Foods from which STECs were recovered included cilantro (6%), spinach (25%), lettuce (11%), and flour (9%). Phylogenetic analysis using core genome MLST revealed these STEC genomes were highly variable, with some clustering associated with ST types and serotypes. We detected 95 different sequence types (ST); several ST were previously associated with HUS: ST21 and ST29 (O26:H11), ST11 (O157:H7), ST33 (O91:H14), ST17 (O103:H2), and ST16 (O111:H-). *in silico* virulome analyses showed ~ 51% of these strains were potentially pathogenic [besides *stx* gene they also carried *eae* (25%) or 26% *saa* (26%)]. Virulence gene prevalence was also determined: *stx*1 only (19%); *stx*2 only (66%); and *stx*1/*sxt*2 (15%). Our data form a new WGS dataset that can be used to support food safety investigations and monitor the recurrence/emergence of *E*. *coli* in foods.

## Introduction

Shiga toxin-producing *Escherichia* coli (STECs) have the potential to cause infections, from mild to life-threatening outcomes such as hemolytic uremic syndrome (HUS). STECs causing HUS, hemorrhagic colitis and bloody diarrhea are known as enterohemorrhagic *E*. *coli* (EHEC). Among the most common EHECs are O157:H7, O26, O121, O103, O111, and O145. O157:H7 strains are responsible for most foodborne outbreaks in the last two decades [1] while non-O157 serogroups, O26, O121, O103, O111, and O145 are the second most common cause of EHEC foodborne infections in the US [2,3] and worldwide [4–7]. Each year in the US, O157:H7 causes approximately 95,000 cases with 2,150 hospitalizations, while non-O157 STECs are responsible for an estimated 170,000 cases [3]. These serotypes carry Shiga toxin genes (*stx1* and/or *stx2*) and there are at least 130 EHEC serotypes that have been recovered from human patients. In 2011 the US Department of Agriculture Food Safety and Inspection Services (USDA FSIS), declared O26 and five other non-O157 serogroups, O45, O103, O111, O121, and O145 as adulterants in ground beef and non-intact beef products, and in mid-2012 began testing for these pathogens in both domestic and imported beef trimmings [8].

In order to cause illness, STEC strains need a set of genes that allow them to attach, colonize, and produce and secrete Shiga toxin protein [9–12]. STECs have the capacity to produce attaching and effacing (A/E) lesions on intestinal mucosa, mediated by proteins found on the locus of enterocyte effacement (LEE) Pathogenicity Island. These include EAE protein (intimin, coded by the *eae* gene), secreted effector proteins (Esp), a TIR receptor, and other T3SS effectors present in the LEE island. However, some STECs do not carry *eae* (LEE-negative STEC strains). They possess other genes believed to compensate for the lack of *eae* or LEE island (e.g. *saa*) and still caused sporadic cases of HUS [13,14]. Other putative virulence genes (*ehxA*, *espP*, *etpD*, *toxB*, *katP*, *subA*, *saa*, and *sab* genes, among others) are usually located in a plasmid referred to as the virulence plasmid to differentiate it from other possible plasmids that can be carried by the same strain [9,11,15]. All STECs have a virulence plasmid, although differing in sizes and gene content (either virulence or plasmid maintenance genes). Although the precise role of *ehxA* in STEC pathogenesis remains to be elucidated, several studies indicate an association of *ehxA* in clinical disease since 1) *ehxA* was found to be produced by many STEC associated with diarrheal disease and HUS [16–18], and 2) serum samples from HUS patients have been shown to react specifically to *ehxA* [19].

STECs can be transmitted by various means with food remaining the predominant transmission route [1]. Among the illnesses caused by STECs in FDA regulated food products (FRFDA), fresh produce has been implicated in several outbreaks, as well as some other atypical commodities, such as flour [20]. Leafy greens and other agricultural food crops are particularly susceptible to contamination since they are grown in close contact with the ground where runoff from livestock areas, particularly cattle, contaminated irrigation water, manure used as fertilizer, and the intrusion of wildlife into growing fields can occur [21]. Many of these same items are consumed raw and possibly with little cleaning. Noteworthy *E*. *coli* outbreaks reported by the Center of Disease and Control (CDC) in the US in the last 10 years are: in 2009—beef (O157:H7) and prepackaged cookie dough (O157:H7); in 2010—cheese (O157:H7), romaine lettuce (O145) and beef (O157:H7); in 2011—romaine lettuce (O157:H7), Lebanon bologna (O157:H7), and in-shell hazelnuts (O157:H7); in 2012—spinach and spring mix blend (O157:H7), unknown source (O145), and raw clover sprouts (O26); in 2013—ready-to-eat salads (O157:H7), and frozen food products (O121); in 2014—raw clover sprouts (O121), and ground beef (O157:H7); in 2015—rotisserie chicken salad (O157:H7), and Mexican-style restaurant chain (O26); in 2016—flour (O121 and O26), and alfalfa sprouts (O157); 2017—leafy greens (O157:H7), and soy nut butter (O157:H7); and in 2018—there has been an outbreak linked to romaine lettuce caused by O157:H7 (https://www.cdc.gov/ecoli/outbreaks.html).

Beyond the noted outbreaks, there have been several reports on STECs found in FRFDA [22,23]. The most comprehensive survey was the USDA Microbiological Data Program (MDP) that collected domestic and imported fresh fruit and vegetable samples from primarily terminal markets and wholesale distribution centers from 2001–2012 (https://www.ams.usda.gov/datasets/mdp/mdp-program-data-and-reports). This program tested approximately 15,000 samples annually, and tested for the presence of *Salmonella*, *E*. *coli O157*:H7, and other STECs. STEC were most frequently found in spinach samples (0.5%), and of the 132 isolated STECs, 9% were found to carry *eae*. The most prevalent Shiga toxin variants found were *stx*1a (22%) and/or *stx*2a (56%) [23]. However, little other information about the genome content of those strains is publicly available.

Whole genome sequencing (WGS) technology is reshaping food safety and food-borne illness investigations [24]. The use of WGS is becoming more useful as the cost of bacterial genome sequencing decreases every year. WGS’s cost per bacterial sequence is now comparable to PFGE. There are many attractive attributes with regards to the use of WGS in analyzing food samples including the potential to identify all pathogens present in that sample [25]. Other applications of WGS may include assistance in the following: identifying genes that allow for resistance/survival or virulence of certain bacterial strains [26–28], establishing phylogenetic relationships among old strains of STECs isolated from either clinical cases or environmental samples [7,29,30], and identifying matches between environmental and outbreak strains during outbreak scenarios [26,30–32]. Furthermore, using WGS can help in identifying matches among bacterial strains isolated from environmental samples in production facilities and locating contamination sources [33]. It can also be extremely helpful in establishing a mechanism of evolution among pathogens [34]. For example, the 2011 outbreak in Germany was linked to fenugreek seeds caused by an *E*. *coli* strain with a genomic backbone and virulence traits of entero-aggregative *E*. *coli* (EAEC) but had acquired a *stx* phage (*stx*2a gene variant) and caused a more aggressive disease with high HUS rate cases [31,35]. This event highlighted the high plasticity of the *E*. *coli* genomes and it constitutes a warning of the possible rise of new “hybrid pathotype” strains.

Therefore, we wanted to further characterize and catalog historical strains of STECs isolated from FRFDA by performing WGS analysis of every STEC strain isolated by the MDP and other FDA surveillance programs, as well as some FDA historical isolates. Most STEC strains were isolated during active surveillance programs and were not outbreak related with the exception of some strains isolated during a flour outbreak of 2016 (ST155 –O121:H19). This work establishes the first genomic dataset of FRFDA STEC strains, which in turn, will allow improved surveillance for both recurrence and the emergence of new strains that may impact our food supply. A total of 296 presumptive STECs were isolated during 2010–2017, and 35 additional STECs were historical isolates from our collection. The 331 presumptive STEC strains were analyzed for virulence genes [encompassing all *E*. *coli* virulent types—STEC, entero-pathogenic *E*. *coli* (EPEC), entero-toxigenic *E*. *coli* (ETEC), entero-invasive *E*. *coli* (EIEC), and EAEC], *in silico* MLST, and antibiotic resistance genes. Finally, their phylogenetic relationships and diversity were determined by whole genome phylogeny analysis using an allele-based whole genome multilocus sequence analysis (MLST) or core genome MLST analysis (cgMLST).

## Materials and methods

### Bacterial strains and media

*E*. *coli* (n = 331) presumptive Shiga toxin-positive strains used in this study are listed in S1 Table. Each strain was assigned a CFSAN number for future tracking. The FRFDA strains were isolated by MDP program (n = 196), FDA Office of Regulatory Affairs (ORA) laboratories (n = 74) during food surveillance testing, and a contracting lab (n = 63) during 2012–2017 in the US.

### DNA preparation

Genomic DNA from each strain was isolated from overnight cultures using the DNeasy Blood and Tissue Kit (QIAGEN, Valencia, CA), following the manufacturer’s instructions. The resultant DNA extract was stored at -20°C until used as a template for whole genome sequencing. The concentration was determined using a Qubit double-stranded DNA HS assay kit and a Qubit 2.0 fluorometer (Thermo Fisher Scientific, Waltham, MA), according to the manufacturer's instructions.

### Whole genome sequencing, contig assembly and annotation

The genomes of the strains sequenced in our laboratory used an Illumina MiSeq sequencer (Illumina, San Diego, CA), with the 2x250 bp pair-end chemistry according to the manufacturer’s instructions, at approximately 80X average coverage. The genome libraries were constructed using the Nextera XT DNA sample prep kit (Illumina). Genomic sequence contigs were *de novo* assembled using default settings within CLC Genomics Workbench v9.5.2 (QIAGEN) with a minimum contig size threshold of 500 bp in length.

### *in silico* MLST phylogenetic analysis

The initial analysis and identification of the strains were performed using an *in silico E*. *coli* MLST approach, based on the information available at the *E*. *coli* MLST website (http://mlst.warwick.ac.uk/mlst/dbs/Ecoli) and using Ridom SeqSphere+ software v2.4.0 (Ridom; Münster, Germany) (http://www.ridom.com/seqsphere). Seven housekeeping genes (*dnaE*, *gyrB*, *recA*, *dtdS*, *pntA*, *pyrC*, and *tnaA*), described previously for *E*. *coli* [36], were used for MLST analysis. The same *E*. *coli* MLST database was also used to assign numbers for alleles and STs.

### *in silico* serotyping, determination of virulence genes, and antimicrobial resistance genes identification

We used Ridom software for performing batch screening of determinants for each of the sequenced genomes.

The serotype of each strain analyzed in this study was confirmed using the genes deposited in the Center for Genomic Epidemiology (http://www.genomicepidemiology.org) for *E*. *coli* as part of their web-based serotyping tool (SerotypeFinder 1.1 - https://cge.cbs.dtu.dk/services/SerotypeFinder) [37,37,38]. Each whole genome sequence was screened for O-type or H-type genes. The virulence genes present in each strain were determined using the genes deposited in the Center for Genomic Epidemiology (http://www.genomicepidemiology.org) for *E*. *coli* as part of their VirulenceFinder 1.5 web-based tool (https://cge.cbs.dtu.dk/services/VirulenceFinder) [38]. Each WGS was screened for the presence of 102 virulence genes (95 virulence genes previously reported here [27] plus 7 additional genes) (S2 Table). These 102 virulence genes included a repertoire representing genes found in different *E*. *coli* pathotypes (ETEC, STEC, EAEC, and EPEC) in order to detect any possible *E*. *coli* hybrid present. Antimicrobial resistance genes present in each sequenced genomes were identified by using the genes deposited in the Center for Genomic Epidemiology (http://www.genomicepidemiology.org) as part of their Resfinder 2.1 web-based tool (https://cge.cbs.dtu.dk/services/ResFinder) [39]. Each WGS was screened for the presence of those AMR genes reported in the database.

### Phylogenetic relationship of the strains by cgMLST analysis

The phylogenetic relationship of the strains was assessed by a core genome multilocus sequence typing (cgMLST) analysis using Ridom SeqSphere+ software v2.4.0. The genome of O157:H7 strain Sakai (NC_002695.1) was used as the reference for the cgMLST. This *E*. *coli* strain has 5,204 genes, of which 3,860 genes (core genes) were present in the six genomes used as comparison to generate the cgMLST scheme (NC_011353.1 –O157:H7 strain EC4115, NC_002655.2—O157:H7 strain EDL933, NC_013008.1 –O157:H7 strain TW14359, NC_013941.1—O55:H7 strain CB9615, NC_017656.1—O55:H7 strain RM12579, and NC_017906.1 –O157:H7 strain Xuzhou21). While 791 genes were found in some of the compared genomes, the remaining of the genes were eliminated from the analysis since they were paralogous or pseudogenes. Therefore, a total of 4,651 genes were used as templates for the analysis of the STECs from this study. After eliminating loci that were missing from the genome of any strain used in our analyses, we performed a cgMLST analysis. These remaining loci were considered the core genome shared by the analyzed strains. We used Nei’s DNA distance method [40] for calculating the matrix of genetic distance, taking into consideration only the number of same/different alleles in the core genes. A Neighbor-Joining (NJ) tree using the appropriate genetic distances was built after the cgMLST analysis. The discriminatory index was calculated with the Ridom software using the Simpson’s discriminatory index as described [41]; cgMLST uses the alleles number of each loci for determining the genetic distance and builds the phylogenetic tree. The use of allele numbers reduces the influence of recombination in the dataset studied and allows for fast clustering determination of genomes.

### Nucleotide sequence accession numbers

The draft genome sequences of 331 *E*. *coli* strains used in our study are available in GenBank under the accession numbers listed in S1 Table.

## Results

### Presence of STEC in FDA regulated foods

Among 331 suspected STEC strains isolated from FRFDA between 2003–2017 and sequenced by several labs and deposited at NCBI, only 276 were confirmed to be STECs by *in silico* analysis for the presence of either *stx1* or *stx2* (S1 Table). Those that were *stx* negatives either might have lost their phages or were initially misidentified. Of the 196 identified by MDP and sequenced by our lab, 92% carried either *stx*1 or 2 (181/196). Of the 74 strains which genomes were retrieved from NCBI and were initially isolated and sequenced by FDA ORA, 94% carried either *stx*1 or 2 (70/74). Of the 63 *E*. *coli* strains isolated and sequenced by a FDA contracting laboratory, 43% carried either stx1 or 2 (25/61). The frequency of isolation of STECs from foods are listed in Table 1. STECs were isolated from 22 food commodities. Most of these STECs were isolated from spinach (32%), flour (21%), lettuce (13%), and cilantro (12%).

**Table 1:** Frequency of STEC isolated by food commodity.

Commodities	No. strains	%
Spinach	88	31.88
Flour	59	21.38
Lettuce	35	12.68
Cilantro (coriander)	32	11.59
Cheese	9	3.26
Leafy greens	8	2.90
Kale	7	2.54
Basil	6	2.17
Pepper	6	2.17
Alfalfa sprouts	3	1.09
Cantaloupe	3	1.09
Parsley	3	1.09
Tomatoes	3	1.09
Creamy soy Nut butter	2	0.72
pizza dough dry mix	2	0.72
sprouts	2	0.72
Almond	1	0.36
oats animal feed	1	0.36
Celery	1	0.36
Clover sprouts	1	0.36
cucumbers	1	0.36
animal feed	1	0.36
enviromental	2	0.72
**Total**	276	100.00

The *stx*-negative strains were eliminated from this analysis.

The frequency of STEC isolation per year, their sequence type, food commodity and state of isolation (if available) is listed in Table 2. The minimum number of STECs recovered from FDA regulated foods per year during the period 2010–2017 was 12 in 2012 and the maximum was 53 in 2016, for a median of 30 STECs per year. We used 2010 as our starting year, since the number of strains prior to that year were found sporadically and came from our STEC historical collection. The STEC analyzed strains were isolated in 22 states.

**Table 2:** Frequency of STECs isolated from FDA regulated food commodities per year from 2010–2017.

Year^a^	Number of STECs Isolated	%	STs	Commodities	State
2003	3	1.09	3017, 641, 446	lettuce, celery, tomato	TX
2004	4	1.45	655, 205, 642	lettuce, cantaloupe, cilantro	TX, CA, MD
2005	1	0.36	677	lettuce	CA
2006	4	1.45	33,211,764,496	alfalfa sprouts, lettuce	MI, CA, MN
2007	2	0.72	297, 295	cantaloupe, lettuce	NY, OH
2008	10	3.62	21, 329, 6475, 642, 2217, 4496, 2008, 1385	spinach, lettuce	CA, WA, MI
2009	11	3.99	223, 58, 11, 6509, 718, 4496, 2389, 6638, 2008, 5530	spinach, lettuce, flour	FL, CA, OH, MI, TX, MD, WI, NY
2010	34	12.32	6640, 2520, 154, 661, 443, 205, 718, 173, 2387, 10, 706, 692, 4173, 1431, 4496, 5299, 3, 295, 5435, 2008, 1727	spinach, lettuce, cilantro, sprouts, hot pepper, tomato	TX, CA, FL, WI, NY, CO, WA,
2011	38	13.77	6642, 942, 297, 6641, 955, 11, 679, 6639, 2161, 21, 692, 2217, 4496, 88, 306, 5435, 205, 2008, 5973, 691, 724	spinach, cantaloupe, lettuce, almond, alfalfa sprouts, cilantro, hot pepper	MI, MN, CA, TX, OH, FL, CO
2012	40	14.49	993, 16, 223, 5975, 2520, 443, 1611, 119, 718, 677, 297, 173, 2387, 101, 21, 5602, 747, 906, 5395, 2385, 4496, 101, 442, 295	cilantro, flour, spinach, lettuce, cherry tomatoes	TX, FL, NC, NY, CO, OH, MI, CA, WA
2013	13	4.71	223, 297, 325, 2388, 1611, 394, 677, 156, 937, 2385, 35, 515	cilantro, basil, sprouts, parsley, flour	GA, CA, TX, TN, AZ
2014	36	13.04	993, 17, 16, 2520, 297, 329, 442, 679, 677, 297, 173, 657, 2387, 21, 342, 43, 906, 675, 6632, 2385, 88, 3759, 2217, 306, 29, 5960, 10	lettuce, kale, cheese, clover sprouts, animal feed, basil, Leafy greens, spinach	OH, CA, WI, AZ, WA, KY, OR, PA
2015	12	4.35	723, 1967, 1817, 25, 398, 32, 442, 205, 446, 21, 342, 40	environmental, flour, lettuce, Leafy greens, spinach, pepper, kale	WA, NE, AZ, CA, OR
2016	53	19.20	655, 747, 723, 4496, 154, 17, 1792, 297, 21, 33	flour, kale, spinach	MO, CO, MI, OK, CA
2017	15	5.43	1112, 5082, 662, 1086, 162	cucumbers, soy nut butter, pepper, flour	KY
**Total**	276	100.00			

^a^- STECs isolated during years 2003–2009 are included as historical STECs and their prevalence was not used for determining the STEC frequency in foods regulated by the FDA per year.

### Characterization of STEC strains by *in silico* MLST

Among the 276 STECs analyzed in this study we identified 95 different sequence types (STs) by *in silico* MLST. Strains belonging to a ST were isolated between 1 to 12 times in the period studied (2010–2017) (Tables 3 and S3). The majority of the STs identified were observed only one time (45%).

**Table 3:** STs observed and number of strains included in each ST. Additionally information is provided for strains belonging to those STs such as: link to human cases, link to EHEC cases, and known serotypes. These additional reports are based on what it is reported in the *E*. *coli* section of the Enterobase database (http://enterobase.warwick.ac.uk).

ST^a^	No. Strains	%	Human cases	Reported as EHEC^b^	Known serotypes
655	38	13.67	+	+	O121:H19
4496	12	4.32	+	NR	O8:H28
2008	12	4.32	+	NR	Ounk:H2/40
21	8	2.88	+	+	O26:H11/-
297	9	3.24	+	- (UPEC, APEC)	diverse serotypes
205	7	2.52	+	- (UPEC)	NR
43	5	1.80	+	- (ETEC, EAEC)	O6:H10
154	5	1.80	+	- (EPEC, APEC)	diverse serotypes
173	5	1.80	+	NR	O181:H49
295	5	1.80	+	- (EAEC, UPEC, ExPEC)	diverse serotypes
677	5	1.80	+	+	diverse serotypes
747	8	2.88	+	- (ETEC)	diverse serotypes
11	3	1.08	+	+	O157:H7/-
16	2	0.72	+	+	O111:H8/2/-
17	2	0.72	+	+	O103:H2/-
25	1	0.36	+	+	O128:H2
29	1	0.36	+	+ (also EPEC)	O26:H11
32	1	0.36	+	+	O145:H-
33	2	0.72	+	+	O91:H14
119	1	0.36	+	+	O165:H25/28
223	4	1.44	+	+ (also UPEC, EAEC)	diverse serotypes
306	2	0.72	+	+	O84:H2/K+
329	2	0.72	+	+ (also EAEC)	diverse serotypes
657	1	0.36	+	+	diverse serotypes
675	1	0.36	+	+	O76:H19
679	3	1.08	+	+	O163:H19
724	1	0.36	+	+	O154/Ounk:H20
5299	2	0.72	+	NR	O8:H49
325	1	0.36	+	NR	O15:H16/K+
6639	1	0.36	+	NR	O174:H21/36
661	2	0.72	+	NR	O174:H2
662	2	0.72	+	NR	diverse serotypes
691	2	0.72	+	NR	diverse serotypes
692	3	1.08	+	NR	O74:H42
718	3	1.08	+	NR	O168:H8
723	4	1.44	+	NR	O103:H11
942	1	0.36	+	NR	O116:H28
955	1	0.36	+	NR	O139:H1/6
993	2	0.72	+	NR	O100:H30
1792	1	0.36	+	NR	O111:H8
1817	1	0.36	+	NR	O104:H7
1967	2	0.72	+	NR	O103:H2
2388	1	0.36	+	NR	O15
2520	3	1.08	+	NR	O116:H49
3759	1	0.36	+	NR	NR
5973	2	0.72	+	NR	Ounk:H2
6475	2	0.72	+	NR	O17/077:H45
10	2	0.72	+	- (mainly EAEC, UPEC, ETEC)	diverse serotypes
35	1	0.36	+	- (EPEC or UPEC)	O154:H4, O145:H34/31
40	1	0.36	+	- (EAEC)	O111ac:H21
58	1	0.36	+	- (EAEC, UPEC, ExPEC, APEC)	diverse serotypes
88	2	0.72	+	- (EAEC, UPEC, ExPEC, APEC)	diverse serotypes
101	3	1.08	+	- (EAEC, UPEC, ExPEC, APEC)	diverse serotypes
156	1	0.36	+	- (UPEC, EXPEC)	diverse serotypes
162	1	0.36	+	- (UPEC, APEC)	O8:H19
342	2	0.72	+	- (EPEC)	O177:NM
394	1	0.36	+	- (EAEC, UPEC)	diverse serotypes
398	1	0.36	+	- (ExPEC)	diverse serotypes
442	3	1.08	+	- (EPEC)	O146:H21
443	2	0.72	+	- (UPEC)	NR
446	2	0.72	+	- (APEC)	diverse serotypes
515	1	0.36	+	- (EAEC)	O2:H9
641	1	0.36	+	- (ExPEC)	diverse serotypes
642	3	1.08	+	- (EPEC)	diverse serotypes
706	1	0.36	+	- (UPEC)	diverse serotypes
906	3	1.08	+	- (UPEC)	diverse serotypes
1431	1	0.36	+	- (ExPEC)	O8:H19/30
1727	4	1.44	+	- (mostly nonpathogen)	diverse serotypes
937	1	0.36	+	- (ExPEC, non pathogen)	O43:H2
1385	1	0.36	-	- (APEC)	Ounk:H4
1611	2	0.72	-	- (APEC)	diverse serotypes
5082	1	0.36	-	NR	NR
332	1	0.36	-	NR	O171:H2
5395	1	0.36	-	NR	O74:H8
5435	2	0.72	-	NR	Ounk:H16
5530	1	0.36	-	NR	Ounk:H21
5602	2	0.72	-	NR	36:H28
5960	1	0.36	-	NR	NR
5975	1	0.36	-	NR	O113:H21
6509	1	0.36	-	NR	O168:H8
6632	1	0.36	-	NR	O8:H16
6638	1	0.36	-	NR	Ounk:H19
3017	1	0.36	-	NR	O116:H21
6640	1	0.36	-	NR	O113:H21
6641	1	0.36	-	NR	O130:H11
6642	1	0.36	-	NR	O113:H21
1112	1	0.36	-	- (nonpathogen)	diverse serotypes
1176	1	0.36	-	- (nonpathogen)	O36:H14
2161	1	0.36	-	- (nonpathogen)	O180:H14
2217	4	1.44	-	- (nonpathogen)	diverse serotypes
2389	1	0.36	-	- (nonpathogen)	NR
1086	10	3.60	-	- (nonpathogen)	diverse serotypes
2385	7	2.52	-	- (nonpathogen)	O8:H19
2387	8	2.88	-	- (nonpathogen)	O185:H7
4173	2	0.72	-	- (nonpathogen)	O79:H2

NR- not reported, UPEC (uropathogenic *E*. *coli*), EPEC (enteropathogenic *E*. *coli*), ETEC (Enterotoxigenic *E*. *coli*), APEC (Avian pathogenic *E*. *coli*), EAEC (Enteroaggregative *E*. *coli*) and ExPEC (Extraintestinal pathogenic *E*. *coli*).

^a^-Determined by *in silico* analysis of the WGS assemblies.

^b^-when negative, the reported *E*. *coli* type is stated.

### Characterization of STEC strains by serotyping, and virulence gene profiles

However, a strain belonging to a known ST that caused hemorrhagic colitis (HC) is not enough to predict the likelihood of it causing disease illness. Therefore, we further characterized these STECs by *in silico* virulence determination and their predicted serotype (Table 4). The detailed in silico analysis for presence of virulence genes and serotype is listed in S2 Table. Table 4 lists only the serotype and some of the most known virulence genes for each strain: *stx1* variants, *stx2* variants, *eae* variants, *ehxA*, *espP*, *etpD*, *toxB*, *katP*, *subA*, *saa*, and *sab*. We identified at least 81 different serotypes among the 276 STECs sequenced (Table 4). Many of the O types were not present in our O types database and were listed as unknown or the wzx and wzy gene were not assembled. Among those some of the most common clinical STECs serotypes were identified, such as: O157:H7, O26:H11, O113:H21, O121:H19, O91:H21, O103:H2, and O111:H8. Other serotypes like O153/O178:H19 could not be discerned by *in silico* analysis since both O types have identical *wzx* and *wzy* gene sequences. This issue is discussed in more detail elsewhere [37].

**Table 4:** *in silico* characterization of STECs from this study for presence of virulence genes and their serotype.

strains	ST	serotype	*stx*1 type	*stx*2 type	eae type	*ehxA*	*espP*	*etpD*	*toxB*	*katP*	*subA*	*saa*	*sab*
CFSAN041120	10	O2:H27	-	a	-	+	-	-	-	-	-	-	-
CFSAN051538	10	Ounk:H32	a	-	-	-	+	-	-	-	-	-	-
CFSAN046715	11	O157:H7	-	a	gamma-1	+	+	+	+	+	-	-	-
CFSAN046720	11	O157:H7	-	a	gamma-1	+	+	+	+	+	-	-	-
FDA00009839	11	O157:H7	-	a	gamma-1	+	+	+	-	+	-	-	-
CFSAN053336	16	O111:H8	a	-	theta-2	+	-	-	-	-	-	-	-
CFSAN053346	16	O111:H8	a	a	theta-2	+	-	-	-	-	-	-	-
FDA00010429	17	O103:H2	a	-	epsilon	+	-	+	-	-	-	-	-
IEH-NGS-ECO-00075	17	O103:H2	a	-	epsilon	+	+	-	+	-	-	-	-
CFSAN046724	21	O26:H11	a	-	beta-1	+	+	-	-	+	-	-	-
CFSAN046724	21	O26:H11	a	-	beta-1	+	-	-	-	+	-	-	-
CFSAN053342	21	O103:H11	a	-	beta-1	+	+	-	+	+	-	-	-
CFSAN053343	21	O26:H11	a	-	beta-1	+	+	-	+	+	-	-	-
CFSAN053345	21	O26:H11	a	-	beta-1	+	+	-	+	+	-	-	-
FDA00010430	21	O26:H11	a	-	beta-1	-	+	-	-	+	-	-	-
IEH-NGS-ECO-00076	21	O26:H11	a	-	beta-1	+	+	-	+	+	-	-	-
IEH-NGS-ECO-00213	21	O26:H11	a	-	beta-1	+	+	-	+	-	-	-	-
IEH-NGS-ECO-00227	25	O128ac:H2	c	-	-	-	-	-	-	-	+	-	+
IEH-NGS-ECO-00125	29	Ounk:H11	-	a	beta-1	+	+	-	-	-	-	-	-
IEH-NGS-ECO-00224	32	O145:H28	a	d	gamma-1	+	+	-	+	+	-	-	-
CFSAN051773	33	O91:H14	a	d	-	+	-	-	-	-	+	+	+
CFSAN053344	33	O91:H14	a	d	-	+	-	-	-	-	+	-	+
IEH-NGS-ECO-00232	40	Ounk:H21	c	-	-	-	-	-	-	+	-	-	-
CFSAN051526	43	O6:H10	c	-	-	-	-	-	-	+	-	-	-
CFSAN051527	43	O6:H10	c	-	-	-	-	-	-	+	-	-	-
CFSAN051533	43	O6:H10	c	-	-	-	-	-	-	+	-	-	-
CFSAN051535	43	O6:H10	c	-	-	-	-	-	-	-	-	-	-
CFSAN051537	43	O6:H10	c	-	-	-	-	-	-	+	-	-	-
CFSAN046659	58	O116:H21	-	a	-	+	+	-	-	-	+	+	+
CFSAN046700	88	O8:H9	-	e	-	-	-	-	-	-	-	-	-
CFSAN051531	88	O8:H30	-	e	-	-	-	-	-	-	-	-	-
CFSAN046737	101	O82:H8	-	a	-	+	+	-	-	-	-	+	+
CFSAN046738	101	O82:H8	-	a	-	+	+	-	-	-	-	+	+
CFSAN046749	101	O21:H21	-	a	-	+	-	-	-	-	+	+	+
CFSAN046750	119	O165:H28	a	a	epsilon-2	+	+	-	-	+	-	-	-
CFSAN046672	154	O88:H25	a	a	-	+	-	-	-	-	-	+	+
CFSAN046673	154	O88:H25	a	a	-	+	-	-	-	-	-	+	+
CFSAN046682	154	O134:H38	a	d	-	+	-	-	-	-	-	+	+
CFSAN056112	154	O88:H25	a	a	-	+	-	-	-	-	-	+	+
CFSAN056113	154	O88:H25	a	a	-	+	-	-	-	-	-	+	+
CFSAN051557	156	O174:H28	-	d	-	-	-	-	-	-	-	-	-
FDA00011520	162	O8:H19	-	d	-	-	-	-	-	-	-	-	-
CFSAN046678	173	O181:H49	a	a	-	+	+	-	-	-	+	+	+
CFSAN046740	173	O181:H49	-	d	-	+	+	-	-	-	+	+	+
CFSAN046747	173	O181:H49	-	d	-	+	+	-	-	-	+	+	+
IEH-NGS-ECO-00108	173	O181:H49	-	a	-	+	-	-	-	-	+	+	+
CFSAN046751	173	O181:H49	-	d	-	+	-	-	-	-	+	+	+
CFSAN041109	205	Ounk:H19	-	a	-	+	-	-	-	-	+	+	+
CFSAN046650	205	O153/O178:H19	-	a	-	+	+	-	-	-	+	+	+
CFSAN046690	205	O153/O178:H19	a	a	-	+	+	-	-	-	+	+	+
CFSAN046704	205	Ounk:H19	-	a	-	+	+	-	-	-	+	+	+
CFSAN051550	205	Ounk:H19	-	a	-	+	+	-	-	-	+	+	+
CFSAN051551	205	Ounk:H19	-	a	-	+	+	-	-	-	+	+	+
FDA00009731	205	O153/O178:H19	a	d	-	+	-	-	-	-	-	+	+
CFSAN046660	223	O113:H21	-	a	-	+	+	-	-	-	+	+	+
CFSAN046665	223	O113:H21	-	a	-	+	+	-	-	-	+	+	+
CFSAN053329	223	O113:H21	-	a	-	+	+	-	-	-	+	+	+
CFSAN046734	223	O113:H21	-	a	-	+	+	-	-	-	+	+	+
CFSAN041114	295	Ounk:H16	-	a	-	+	-	-	-	-	+	+	+
CFSAN046640	295	Ounk:H16	c	b	-	-	-	-	-	-	-	-	+
CFSAN046691	295	Ounk:H11	-	a	-	+	+	-	-	-	+	+	+
CFSAN053347	295	Ounk:H16	-	a	-	+	+	-	-	-	+	+	+
CFSAN046735	295	Ounk:H11	-	a	-	+	-	-	-	-	+	+	+
CFSAN041108	297	O130:H11	a	d	-	+	-	-	-	-	+	+	+
CFSAN046639	297	O130:H11	-	a	-	+	-	-	-	-	+	+	+
CFSAN046719	297	O130:H11	-	d	-	+	-	-	-	-	+	+	+
CFSAN046753	297	O179:H8	-	a	-	+	+	-	-	-	+	+	+
CFSAN051516	297	O130:H11	-	a	-	+	-	-	-	-	+	+	+
CFSAN051524	297	O130:H11	-	a	-	+	-	-	-	-	+	+	+
CFSAN051549	297	O130:H11	a	d	-	+	-	-	-	-	+	+	+
CFSAN056111	297	O130:H11	-	a	-	+	-	-	-	-	+	+	+
IEH-NGS-ECO-00087	297	O179:H8	-	a	-	+	-	-	-	-	+	+	+
CFSAN046717	306	O98:H21	a	-	zeta	+	+	-	-	-	-	-	-
CFSAN051525	306	O98:H21	a	-	zeta	+	+	-	-	-	-	-	-
CFSAN051544	325	O15:H16	-	g	-	-	-	-	-	-	-	-	-
CFSAN046643	329	O136:H16	a	-	-	+	-	-	-	-	-	-	-
CFSAN053337	329	O136:H16	a	-	-	+	-	-	-	-	-	-	-
CFSAN046636	332	O171:H2	-	c	-	-	-	-	-	-	-	-	-
CFSAN053335	342	O5:Hunk	a	-	beta-1	+	+	-	-	-	-	-	-
IEH-NGS-ECO-00230	342	O5:Hunk	a	-	beta-1	+	-	-	-	-	-	-	-
CFSAN053330	394	O17/O77:H18	-	d	-	+	+	-	-	-	+	+	+
IEH-NGS-ECO-00231	398	O136:H20	c	-	-	-	-	-	-	+	-	-	-
CFSAN046746	442	O91:H21	-	a	-	+	-	-	-	-	-	+	+
CFSAN051529	442	O146:H21	c	b	-	+	-	-	-	-	+	-	+
CFSAN051540	442	O146:H21	c	-	-	+	-	-	-	-	+	-	+
CFSAN046688	443	O153/O178:H19	a	d	-	+	-	-	-	-	-	+	+
CFSAN046752	443	O153/O178:H19	a	d	-	-	-	-	-	-	-	+	+
CFSAN046631	446	O22:H8	-	c	-	-	-	-	-	-	-	-	+
FDA00009425	446	O22:H8	-	c	-	-	-	-	-	-	-	-	-
CFSAN053334	515	Ounk:H29	-	d	-	-	-	-	-	-	-	-	-
CFSAN046632	641	O117:H10	a	-	-	-	+	-	-	-	-	+	-
CFSAN046646	642	O187:H52	c	-	-	-	-	-	-	-	-	-	-
CFSAN046648	642	O187:H52	c	-	-	-	-	-	-	-	-	-	-
CFSAN046649	642	O187:H52	c	-	-	-	-	-	-	-	-	-	-
CFSAN046651	655	O121:H19	-	a	epsilon-2	+	-	-	-	-	-	-	-
FDA00010253	655	O121:H19	-	a	epsilon-2	+	+	-	-	-	-	-	-
FDA00010254	655	O121:H19	-	a	epsilon-2	+	+	-	-	-	-	-	-
FDA00010255	655	O121:H19	-	a	epsilon-2	+	+	-	-	-	-	-	-
FDA00010256	655	O121:H19	-	a	epsilon-2	-	-	-	-	-	-	-	-
FDA00010257	655	O121:H19	-	a	epsilon-2	+	+	-	-	-	-	-	-
FDA00010258	655	O121:H19	-	a	epsilon-2	-	-	-	-	-	-	-	-
FDA00010259	655	O121:H19	-	a	epsilon-2	-	-	-	-	-	-	-	-
FDA00010276	655	O121:H19	-	a	epsilon-2	-	-	-	-	-	-	-	-
FDA00010277	655	O121:H19	-	a	epsilon-2	+	+	-	-	-	-	-	-
FDA00010278	655	O121:H19	-	a	epsilon-2	-	-	-	-	-	-	-	-
FDA00010279	655	O121:H19	-	a	epsilon-2	-	-	-	-	-	-	-	-
FDA00010280	655	O121:H19	-	a	epsilon-2	+	+	-	-	-	-	-	-
FDA00010281	655	O121:H19	-	a	epsilon-2	-	-	-	-	-	-	-	-
FDA00010282	655	O121:H19	-	a	epsilon-2	-	-	-	-	-	-	-	-
FDA00010283	655	O121:H19	-	a	epsilon-2	+	+	-	-	-	-	-	-
FDA00010284	655	O121:H19	-	a	epsilon-2	-	-	-	-	-	-	-	-
FDA00010285	655	O121:H19	-	a	epsilon-2	-	-	-	-	-	-	-	-
FDA00010296	655	O121:H19	-	a	epsilon-2	+	+	-	-	-	-	-	-
FDA00010297	655	O121:H19	-	a	epsilon-2	-	-	-	-	-	-	-	-
FDA00010298	655	O121:H19	-	a	epsilon-2	-	-	-	-	-	-	-	-
FDA00010299	655	O121:H19	-	a	epsilon-2	+	+	-	-	-	-	-	-
FDA00010300	655	O121:H19	-	a	epsilon-2	+	+	-	-	-	-	-	-
FDA00010301	655	O121:H19	-	a	epsilon-2	-	+	-	-	-	-	-	-
FDA00010302	655	O121:H19	-	a	epsilon-2	-	-	-	-	-	-	-	-
FDA00010303	655	O121:H19	-	a	epsilon-2	+	+	-	-	-	-	-	-
FDA00010304	655	O121:H19	-	a	epsilon-2	+	+	-	-	-	-	-	-
FDA00010305	655	O121:H19	-	a	epsilon-2	-	-	-	-	-	-	-	-
FDA00010306	655	O121:H19	-	a	epsilon-2	-	-	-	-	-	-	-	-
FDA00010307	655	O121:H19	-	a	epsilon-2	+	+	-	-	-	-	-	-
FDA00010308	655	O121:H19	-	a	epsilon-2	-	-	-	-	-	-	-	-
FDA00010309	655	O121:H19	-	a	epsilon-2	-	-	-	-	-	-	-	-
FDA00010310	655	O121:H19	-	a	epsilon-2	-	+	-	-	-	-	-	-
FDA00010369	655	O121:H19	-	a	epsilon-2	+	+	-	-	-	-	-	-
FDA00010370	655	O121:H19	-	a	epsilon-2	-	+	-	-	-	-	-	-
FDA00010371	655	O121:H19	-	a	epsilon-2	+	+	-	-	-	-	-	-
FDA00010372	655	O121:H19	-	a	epsilon-2	+	+	-	-	-	-	-	-
FDA00010373	655	O121:H19	-	a	epsilon-2	-	-	-	-	-	-	-	-
IEH-NGS-ECO-00103	657	O183:H18	a	d	-	+	+	-	-	-	+	+	+
CFSAN041119	661	O174:H2	-	c	-	+	+	-	-	-	-	+	+
CFSAN051547	661	O174:H2	-	c	-	+	-	-	-	-	-	+	+
FDA00011331	662	O17/O77:H45	a	d	-	+	+	-	-	-	-	+	+
FDA00011332	662	O17/O77:H45	a	d	-	+	+	-	-	-	-	+	+
CFSAN051530	675	O76:H19	c	-	-	+	-	-	-	-	+	-	+
CFSAN046652	677	Ounk:H21	-	d	-	-	-	-	-	-	-	-	-
CFSAN046748	677	O174:H21	a	d	-	+	+	-	-	-	+	+	+
CFSAN051519	677	O174:H21	-	a	-	-	-	-	-	-	-	-	+
CFSAN051520	677	O174:H21	-	a	-	-	-	-	-	-	-	-	+
IEH-NGS-ECO-00080	677	O174:H21	-	d	-	-	-	-	-	-	-	-	-
CFSAN046699	679	O163:H19	-	d	-	+	+	-	-	-	+	+	+
CFSAN046705	679	O163:H19	-	a	-	+	+	-	-	-	+	+	+
CFSAN051541	679	O163:H19	a	d	-	+	+	-	-	-	+	+	+
CFSAN053338	691	Ounk:H20	a	d	-	+	+	-	-	-	-	+	+
CFSAN046703	691	Ounk:H20	a	-	-	+	+	-	-	-	-	+	+
CFSAN046709	691	Ounk:H20	a	-	-	+	+	-	-	-	-	+	+
CFSAN041113	692	O74:H42	a	d	-	+	+	-	-	-	-	+	+
CFSAN051548	692	O74:H42	a	d	-	+	+	-	-	-	-	+	+
CFSAN051553	692	O74:H42	a	d	-	+	+	-	-	-	-	+	+
CFSAN046689	706	O32:H1	-	d	-	-	-	-	-	-	-	-	+
CFSAN046666	718	O168:H8	-	a	-	+	-	-	-	-	-	-	-
CFSAN046693	718	O168:H8	-	d	-	-	-	-	-	-	-	-	-
CFSAN046725	718	O168:H8	-	d	-	-	-	-	-	-	-	-	-
FDA00010428	723	O103:H11	a	-	beta-1	-	-	-	-	+	-	-	-
FDA00010431	723	O103:H11	a	-	beta-1	+	-	-	-	+	-	-	-
FDA00010457	723	O103:H11	a	-	beta-1	+	+	-	-	+	-	-	-
IEH-NGS-ECO-00223	723	O103:H11	a	-	beta-1	+	+	-	+	+	-	-	-
CFSAN046707	724	Ounk:H20	a	-	-	+	+	-	-	-	+	+	+
CFSAN046733	747	O17/O77:H45	-	a	-	+	+	-	-	-	-	+	+
CFSAN046741	747	O17/O77:H45	-	a	-	+	+	-	-	-	-	+	+
CFSAN046742	747	O17/O77:H45	-	a	-	+	+	-	-	-	-	+	+
CFSAN046743	747	O17/O77:H45	-	a	-	+	+	-	-	-	-	+	+
FDA00010882	747	O17/O77:H45	-	a	-	+	+	-	-	-	-	+	+
FDA00010883	747	O17/O77:H45	-	a	-	+	+	-	-	-	-	+	+
FDA00010884	747	O17/O77:H45	-	a	-	+	+	-	-	-	-	+	+
FDA00010885	747	O17/O77:H45	-	a	-	+	+	-	-	-	-	+	+
CFSAN041116	906	O74:H8	-	d	-	+	+	-	-	-	+	+	+
CFSAN051555	906	O74:H8	-	d	-	+	+	-	-	-	+	+	+
IEH-NGS-ECO-00096	906	O74:H8	-	a	-	+	+	-	-	-	+	+	+
CFSAN051545	937	O43:H2	a	a	-	+	-	-	-	-	-	+	+
CFSAN046721	942	O116:H28	a	-	-	+	+	-	-	-	-	+	+
CFSAN046713	955	O139:H1	-	e	-	-	-	-	-	-	-	-	-
CFSAN051539	993	O100:H30	-	e	-	-	-	-	-	-	-	-	-
CFSAN046730	993	O100:H30	-	e	-	-	-	-	-	-	-	-	-
FDA00011815	1086	O8:H14	-	d/e	-	-	-	-	-	-	-	-	-
FDA00011816	1086	O8:H14	-	d/e	-	-	-	-	-	-	-	-	-
FDA00011817	1086	O8:H14	-	d/e	-	-	-	-	-	-	-	-	-
FDA00011818	1086	O8:H14	-	d/e	-	-	-	-	-	-	-	-	-
FDA00011819	1086	O8:H14	-	d/e	-	-	-	-	-	-	-	-	-
FDA00011820	1086	O8:H14	-	d/e	-	-	-	-	-	-	-	-	-
FDA00011821	1086	O8:H14	-	d/e	-	-	-	-	-	-	-	-	-
FDA00011822	1086	O8:H14	-	d/e	-	-	-	-	-	-	-	-	-
FDA00011823	1086	O8:H14	-	d/e	-	-	-	-	-	-	-	-	-
FDA00011824	1086	O8:H14	-	d/e	-	-	-	-	-	-	-	-	-
FDA00011218	1112	O142:H27	-	e	-	-	-	-	-	-	-	-	-
CFSAN046653	1176	O36:H14	-	g	-	+	-	-	-	-	-	-	-
CFSAN046655	1385	Ounk:H4	-	d	-	-	-	-	-	-	-	-	-
CFSAN046683	1431	O8:H19	-	d/e	-	+	-	-	-	-	-	-	-
CFSAN046728	1611	O159:H19	-	d/e	-	+	-	-	-	-	-	-	-
CFSAN051515	1611	O159:H19	-	d/e	-	-	-	-	-	-	-	-	-
CFSAN046668	1727	Ounk:H7	-	c	-	-	-	-	-	-	-	-	-
CFSAN046669	1727	Ounk:H7	-	c	-	-	-	-	-	-	-	-	-
CFSAN046671	1727	Ounk:H7	-	c	-	-	-	-	-	-	-	-	-
CFSAN046692	1727	Ounk:H7	-	c	-	-	-	-	-	-	-	-	-
FDA00010432	1792	O111:H8	a	a	theta-2	+	-	-	-	-	-	-	-
IEH-NGS-ECO-00221	1817	O104:H7	c	-	-	-	-	-	-	+	-	-	-
IEH-NGS-ECO-00211	1967	O103:H2	a	-	epsilon	+	+	-	-	+	-	-	-
IEH-NGS-ECO-00212	1967	O103:H2	a	-	epsilon	+	+	-	-	-	-	-	-
CFSAN041110	2008	Ounk:H2	-	d	-	-	-	-	-	-	-	-	-
CFSAN041111	2008	Ounk:H2	-	d	-	-	-	-	-	-	-	-	-
CFSAN041112	2008	Ounk:H2	-	d	-	-	-	-	-	-	-	-	-
CFSAN046642	2008	Ounk:H2	-	a	-	-	-	-	-	-	-	-	-
CFSAN046657	2008	Ounk:H2	-	d/e	-	-	-	-	-	-	-	-	-
CFSAN046684	2008	Ounk:H2	-	d/c	-	-	-	-	-	-	-	-	-
CFSAN046685	2008	Ounk:H2	-	d/c	-	-	-	-	-	-	-	-	-
CFSAN046686	2008	Ounk:H2	-	d/c	-	-	-	-	-	-	-	-	-
CFSAN046687	2008	Ounk:H2	-	d/c	-	-	-	-	-	-	-	-	-
CFSAN046708	2008	Ounk:H2	-	d/c	-	-	-	-	-	-	-	-	-
CFSAN046710	2008	Ounk:H2	-	d/c	-	-	-	-	-	-	-	-	-
CFSAN051552	2008	Ounk:H2	-	d/c	-	-	-	-	-	-	-	-	-
CFSAN046701	2161	O180:H14	-	d/e	-	-	-	-	-	-	-	-	-
CFSAN046641	2217	O45:H16	a	-	-	-	+	-	-	-	-	+	-
CFSAN046712	2217	O76:H21	a	-	-	-	-	-	-	-	-	+	-
CFSAN046714	2217	O8:H16	a	-	-	-	+	-	-	-	-	+	-
CFSAN051522	2217	O84:H38	a	-	-	-	+	-	-	-	-	+	-
CFSAN046723	2385	O8:H19	a	a	-	+	+	-	-	-	+	+	+
CFSAN051517	2385	O8:H19	a	a	-	+	+	-	-	-	+	+	+
CFSAN051518	2385	O8:H19	a	a	-	+	+	-	-	-	+	+	+
IEH-NGS-ECO-00082	2385	O8:H19	a	a	-	+	+	-	-	-	+	+	+
IEH-NGS-ECO-00088	2385	O8:H19	-	a	-	+	+	-	-	-	+	+	+
IEH-NGS-ECO-00089	2385	O8:H19	-	a	-	+	+	-	-	-	+	+	+
IEH-NGS-ECO-00090	2385	O8:H19	a	c	-	+	+	-	-	-	+	+	+
CFSAN041115	2387	O185:H7	-	c	-	-	-	-	-	-	-	-	-
CFSAN041118	2387	O185:H7	-	c	-	-	-	-	-	-	-	-	-
CFSAN046670	2387	O185:H7	-	c	-	-	-	-	-	-	-	-	-
CFSAN051528	2387	O185:H7	-	c	-	+	+	-	-	-	-	-	-
CFSAN051546	2387	O185:H7	-	c	-	+	+	-	-	-	-	-	-
CFSAN051554	2387	O185:H7	-	c	-	-	-	-	-	-	-	-	-
CFSAN046744	2387	O185:H7	-	c	-	-	-	-	-	-	+	+	+
CFSAN046745	2387	O185:H7	-	c	-	-	+	-	-	-	+	+	+
CFSAN053339	2388	O15:H27	-	d	-	-	-	-	-	-	-	-	-
CFSAN046656	2389	Ounk:H11	-	d	-	+	+	-	-	-	+	+	+
CFSAN046679	2520	O116:H49	-	a	-	+	+	-	-	-	+	+	+
CFSAN046731	2520	O116:H49	-	a	-	+	+	-	-	-	+	+	+
IEH-NGS-ECO-00204	2520	O116:H49	a	a	-	+	+	-	-	-	+	+	+
CFSAN046633	3017	O116:H21	a	a	-	+	-	-	-	-	+	+	+
IEH-NGS-ECO-00070	3759	O8:H49	-	a	-	+	-	-	-	-	+	+	+
CFSAN046667	4173	O79:H2	a	-	-	-	-	-	-	-	-	-	-
CFSAN046676	4173	O79:H2	a	-	-	-	-	-	-	-	-	-	-
CFSAN046637	4496	O8:H28	-	d/e	-	+	-	-	-	-	-	-	-
CFSAN046644	4496	O8:H28	-	d/e	-	+	-	-	-	-	-	-	-
CFSAN046654	4496	O8:H28	-	d/e	-	+	-	-	-	-	-	-	-
CFSAN046661	4496	O8:H28	-	d/e	-	+	-	-	-	-	-	-	-
CFSAN046680	4496	O8:H28	-	d/e	-	+	-	-	-	-	-	-	-
CFSAN046695	4496	O8:H28	-	d/e	-	+	-	-	-	-	-	-	-
CFSAN046718	4496	O8:H28	-	d/e	-	+	-	-	-	-	-	-	-
CFSAN046726	4496	O8:H28	-	d/e	-	+	-	-	-	-	-	-	-
CFSAN046727	4496	O8:H28	-	d/e	-	+	-	-	-	-	-	-	-
FDA00009866	4496	O8:H28	-	d/e	-	+	-	-	-	-	-	-	-
FDA00009867	4496	O8:H28	-	d/e	-	-	-	-	-	-	-	-	-
CFSAN046729	4496	O8:H28	-	d/e	-	+	-	-	-	-	-	-	-
FDA00011519	5082	O180:H14	-	d/e	-	-	-	-	-	-	-	-	-
CFSAN046674	5299	O8:H49	a	-	-	-	-	-	-	-	-	+	+
CFSAN046675	5299	O8:H49	a	-	-	+	+	-	-	-	-	+	+
CFSAN046722	5395	O74:H8	a	-	-	+	+	-	-	-	+	+	+
CFSAN046694	5435	Ounk:H16	-	a	-	+	-	-	-	-	+	+	+
CFSAN046696	5435	Ounk:H16	-	a	-	+	+	-	-	-	+	+	+
CFSAN046664	5530	Ounk:H21	-	d/e	-	-	-	-	-	-	-	-	-
CFSAN041117	5602	O36:H28	-	g	-	+	-	-	-	-	-	-	-
CFSAN051556	5602	O36:H28	-	g	-	+	-	-	-	-	-	-	-
IEH-NGS-ECO-00105	5960	Ounk:H19	-	a	-	+	+	-	-	-	+	+	+
CFSAN046702	5973	Ounk:H2	-	d	-	+	+	-	-	-	+	+	+
CFSAN046716	5973	Ounk:H2	-	c	-	+	+	-	-	-	+	+	+
CFSAN046739	5975	O113:H21	-	a	-	+	+	-	-	-	+	+	+
CFSAN046645	6475	O17/O77:H45	a	a	-	+	+	-	-	-	-	+	+
CFSAN046647	6475	O17/O77:H45	a	a	-	+	+	-	-	-	-	-	-
CFSAN046663	6509	O168:H8	-	d/e	-	+	-	-	-	-	-	-	-
CFSAN051521	6632	O8:H16	-	d	-	-	+	-	-	-	-	+	-
CFSAN046662	6638	Ounk:H19	-	a	-	+	-	-	-	-	+	+	+
CFSAN046711	6639	O174:H21	-	c	-	-	+	-	-	-	-	-	-
CFSAN046677	6640	O113:H21	-	d	-	+	+	-	-	-	+	+	+
CFSAN046706	6641	O130:H11	a	-	-	+	-	-	-	-	+	+	+
CFSAN046697	6642	O113:H21	-	a	-	+	+	-	-	-	+	+	+

Adherence factors *eae* and *saa* genes were found in 67 (24%) and 72 (26%), respectively (Table 4). Shiga toxin genes were present as follows: *stx*1- 53 (19%) (variants a and c), *stx*2- 184 (67%) (variants a, b, c, d, d/e, e, and g), while *stx*1+*stx*2–39 (15%). Among the 184 STECs carrying only *stx2* genes, 144 of them carried variants *2a*,*d*, or *c*; while the remaining 40 carried variants *2e*, *d/e*, or *g*.Other putative virulence genes were found as follows: *exhA* gene was present in 169 (61%), *espP* was present in 118 (43%), *katP* in 24 (9%), *etpD* in 4 (2%), and finally *toxB* was present in 10 (4%).

### Presence of antimicrobial resistance genes

Thirty-three of the 276 STEC strains (12%) carried antimicrobial resistance genes (Table 5). Thirty of them carried multiple antibiotic resistance genes while the remaining three carried a single gene (*tetA*- IEH-NGS-ECO-00231, FDA00011218, and CFSAN051521). Among the antimicrobial classes observed were genes resistant to aminoglycosides, beta-lactamases, macrolides, phenicols, quinolones, sulphonamides, tetracyclines, and trimethoprim.

**Table 5:** Presence of antimicrobial resistance genes identified by *in silico* analysis in the 276 STEC genomes analyzed in this study.

Strains	*aadA*^*a*^	*aph3*^*a*^	*strA*^*a*^	*strB*^*a*^	*bla* *TEM*^*b*^	*mef(B)*^c^	*floR*^*d*^	*QnrB*^*e*^	*sul1*^*f*^	*sul2*^*f*^	*sul3*^*f*^	*tetA*^*g*^	*tetB*^*g*^	*tetC*^*g*^	*dfrA*^*h*^
CFSAN053338	-	-	+	+	-	-	-	-	-	-	-	-	-	-	-
CFSAN046714^i^	-	-	-	-	-	-	-	-	+	-	+	-	-	+	-
CFSAN051552	-	-	+	+	-	-	-	-	-	+	-	-	+	-	-
CFSAN046710	-	-	+	+	-	-	-	-	-	+	-	-	+	-	-
CFSAN046642	-	-	+	+	-	-	-	-	-	+	-	-	+	-	-
IEH-NGS-ECO-00231^j^	-	-	-	-	-	-	-	-	-	-	-	+	-	-	-
CFSAN053336	-	+	+	+	-	-	-	-	-	+	-	+	-	-	-
FDA00009425	-	-	+	+	-	-	+	-	-	+	-	+	-	-	-
FDA00011218	-	-	-	-	-	-	-	-	-	-	-	+	-	-	-
IEH-NGS-ECO-	+	-	+	+	-	-	+	-	+	+	+	-	-	-	-
CFSAN051526	+	-	-	-	-	-	-	-	+	-	+	+	-	-	-
CFSAN046636	-	-	+	+	-	-	-	-	-	+	-	-	+	-	-
CFSAN046669	-	-	+	+	-	-	-	-	-	+	-	-	+	-	-
CFSAN046668	-	-	+	+	-	-	-	-	-	+	-	-	+	-	-
CFSAN053334	-	-	+	+	-	-	+	-	-	+	-	+	-	-	-
CFSAN046687	-	-	+	+	-	-	-	-	-	+	-	-	+	-	-
CFSAN051521	-	-	-	-	-	-	-	-	-	-	-	+	-	-	-
CFSAN046730	-	+	+	+	-	-	-	-	-	+	-	-	+	-	-
CFSAN041112	-	-	+	+	-	-	-	-	-	+	-	-	+	-	-
CFSAN041111	-	-	+	+	-	-	-	-	-	+	-	-	+	-	-
CFSAN046671	-	-	+	+	-	-	-	-	-	+	-	-	+	-	-
CFSAN046708	-	-	+	+	-	-	-	-	-	+	-	-	+	-	-
CFSAN046685	-	-	+	+	-	-	-	-	-	+	-	-	+	-	-
CFSAN046684	-	-	+	+	-	-	-	-	-	+	-	-	+	-	-
CFSAN051527	+	-	-	-	-	-	-	-	+	-	+	+	-	-	-
CFSAN046686	-	-	+	+	-	-	-	-	-	+	-	-	+	-	-
CFSAN051535	-	-	+	+	+	-	-	-	-	+	-	+	-	-	-
CFSAN046693	-	-	+	+	-	-	-	-	-	+	-	-	-	-	-
CFSAN046713	-	-	-	+	+	-	-	+	-	+	-	+	-	-	+
CFSAN046725	-	-	-	+	-	-	-	-	-	+	-	-	-	-	-
CFSAN051531	-	-	-	+	-	-	-	-	-	+	-	+	-	-	+
CFSAN051539^k^	+	+	-	-	+	+	-	-	-	-	+	-	+	-	-
CFSAN041110	-	-	+	+	-	-	-	-	-	+	-	-	+	-	-

^a^Aminoglycoside,

^b^Beta-lactamase,

^c^Macrolide,

^d^Phenicol,

^e^Quinolone,

^f^Sulphonamide,

^g^Tetracycline, and

^h^ Trimethoprim.

^i^ strain carrying *blaOXA*^b^ gene.

^j^ strain carrying *blaCMY-2*^*b*^ gene.

^k^ strain carrying *cml*^*d*^ and *cmlA1*^d^ genes.

### Phylogenetic relationship of the STEC strains by cgMLST analysis

**Fig 1:**
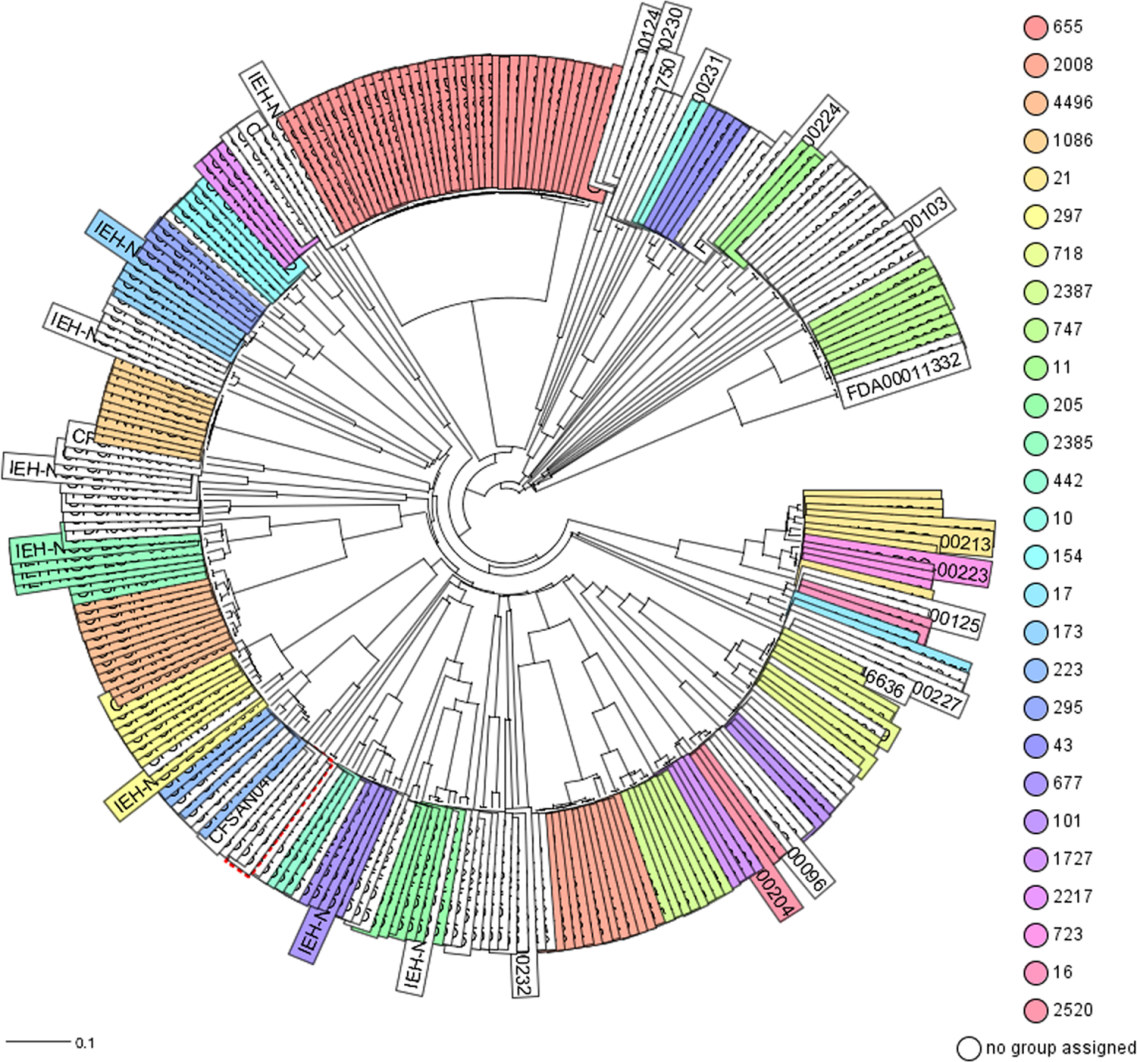
Phylogenetic relationships among the 276 STEC genomes of *E*. *coli* sequenced in this study by cgMLST analysis. Ridom SeqSphere+ (v5.0.0) identified 4,651core genes. The evolutionary history was inferred by using the Neighbor-joining (NJ) tree built using the genetic distance and showing the existence of many diverse clades with a complex evolutionary history. Strains are colored based on different STs as labeled.

**Fig 2:**
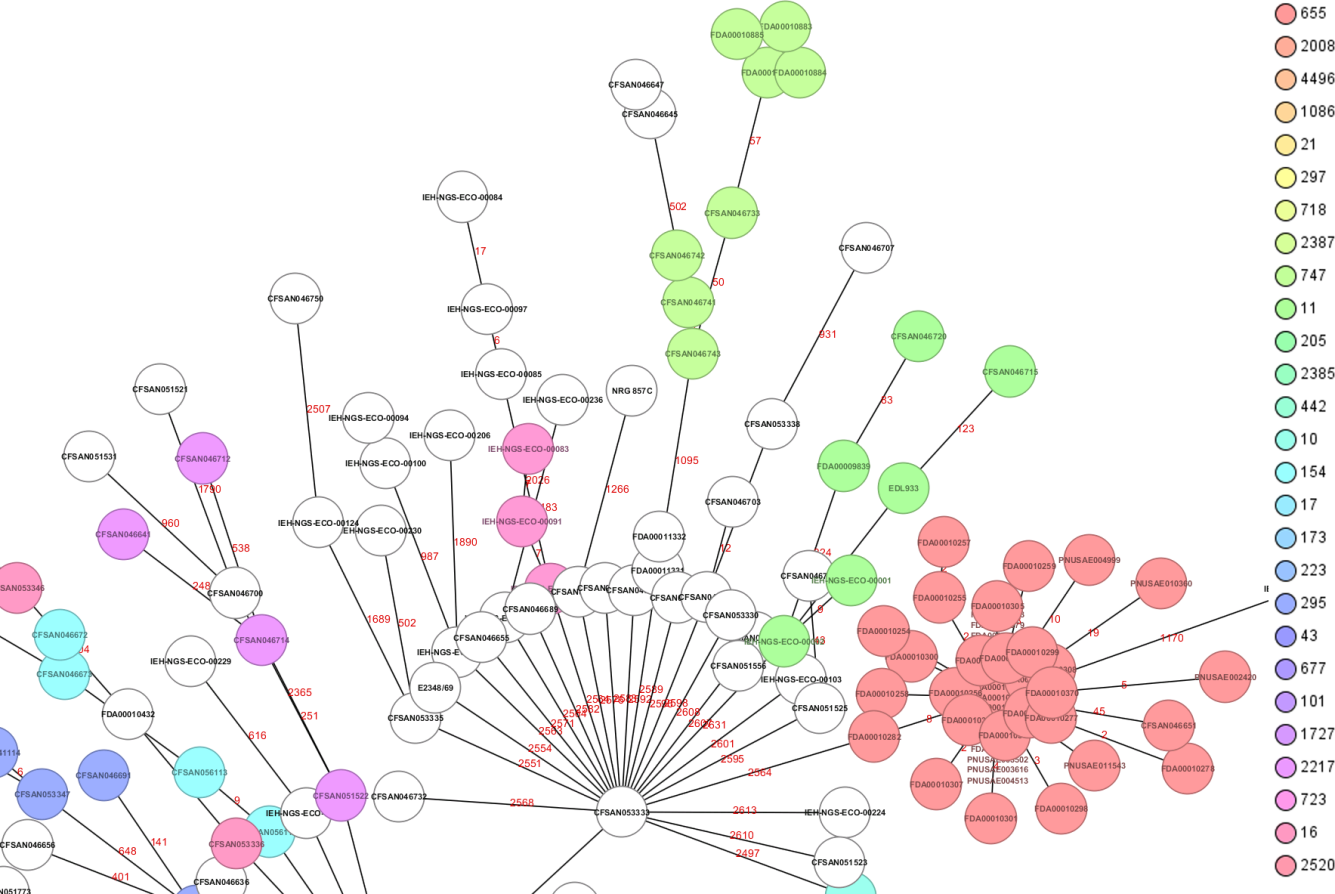
View of a segment from the minimum spanning tree (MST) showing the relationships among all different STECs (complete MST in S1 Fig). The numbers above the connected lines (not to scale) represent allele differences between strains belonging to the same ST. The isolates are colored based on different STs as labeled.

The phylogenetic relationships among the 276 STECs from this study determined by cgMLST analysis is shown in Fig 1. The genome of O157:H7 strain Sakai (NC_002695.1) was used as the reference for the cgMLST. The initial phylogenetic analysis [Neighbor-Joining (NJ) tree] based on gene differences (allele based) among these 276 STECs (Fig 1) revealed a complex evolutionary history with the existence of multiple, highly diverse genomic variants of strains isolated from RFFDA. Some of these genomes formed discrete groups and clustering was consistent with their ST (ex. all ST655 strains clustered together). A further analysis by a minimum spanning tree allows visualization of allele differences between strains with the same ST that was not seen with the NJ tree (Fig 2).

### *eae* positive Non-STEC strains virulence gene profiles

Among the 55 non-STECs (lacking either *stx* gene by *in silico* analysis) strains isolated from FDA regulated foods, we found 35 that were positive for the *eae* gene (S4 Table). The majority were classified as atypical EPEC (aEPEC) *eae*^+^ and *bfpA*^-^. Two of them (IEH-NGS-ECO-00094, and IEH-NGS-ECO-00100) carried *bfpA -*the first gene of the *bfp* operon that encodes a type IV pilus- *(eae*^*+*^ and *bfpA*^*+*^) but were missing most of the common genes found in typical EPEC (S4 Table, typical EPEC lineage 1 strain E2348/69). Therefore, we classified them as aEPEC.

## Discussion

STECs are the most dangerous among diarrheagenic *E*. *coli* affecting public health worldwide [5,7,23,27,42,43]. Usually the most threatening STEC are those of O157:H7 serotype [44,45]. However, in recent years there has been an increase in the occurrence of many non-O157 serotypes in humans associated with consumption of contaminated food, including produce and other FDA regulated products [2,46,47]. Some studies have characterized STECs presence and their virulence potential from FDA regulated products [20,23,48]. Most of the STEC isolated from those products have been only initially screened for the presence of some virulence genes using PCR [23,49]. In the present study, we performed an in-depth analysis with whole genome sequencing of 331 presumptive STEC strains. These strains were isolated from FDA regulated foods recovered during a period of 2003–2017 by two surveillance programs (FDA ORA, and MDP USDA). STECs were isolated from 22 food commodities. It is worth mentioning that even though the sampling did not occur in all states, the food commodities had nationwide (or at least multistate) distribution.

The STEC analyzed in this study were isolated from a wide variety of foods (Table 1), with the majority isolated from spinach (32%), flour (21%), lettuce (13%), and cilantro (12%) samples during the period 2010–2017. The actual frequency of flour STECs should be assessed at a lower frequency of 9%; the spike observed in their frequency was due to the outbreak in flour in 2016, where most STECs (37 strains (66%) of total flour STECs) were isolated. A better reflection of the frequency of STEC isolated per food commodity, specifically produce, can be found in Feng and Reddy (2013) [23]. Nevertheless, the presence of STECs in FRFDA per year remained relatively low, with a median of 30 isolates per year. As pointed previously, these variations in frequency of isolation can be due to seasonal and geographical variations, or to sporadic outbreaks as was observed for O121:H19 STEC strains isolated from flour in 2016 (23).

WGS revealed that these STECs were highly variable with the existence of 95 different sequence types (STs) and belonging to at least 81 different serotypes. Some serotypes could not be predicted and might be due to the fact that the O and H type genes were not present in the database used which includes the most frequent serotypes found in clinical cases. Most STs were observed only once while some others were observed more frequently. ST655 was observed up to 38 times among the STECs analyzed and it was because 37 of those STEC strains were recovered during the flour outbreak in 2016 [20]. According to what was found in Enterobase (http://enterobase.warwick.ac.uk), the majority of the STEC STs observed in this study [69/95–73%] had been reported as causing disease in humans. Furthermore, of these potential human pathogenic STECs, strains belonging to 18 of those STs (19%) were additionally associated with strains causing EHEC-related illnesses (Table 3). Among the known ST associated with causing HC illnesses or HUS cases we found: ST21 and ST29 (O26:H11), ST11 (O157:H7), ST33 (O91:H14), ST17 (O103:H2), and ST16 (O111:H-), among others [7,42,50,51]. There are some STs, that have been described as EAEC/ETEC, however in our dataset, the strains with those STs did not carry any virulence genes for those pathotypes. These results emphasized the need to analyze the data as a whole (e.g. to include virulence genes);ST alone is not an accurate predictor of pathotype or virulence.

Although some strains have the same ST, they show differences in their virulence profile as well as their Shiga toxin gene content. For example, there were 5 strains that were ST10 and from these only 2 were classified as STECs, with one carrying *stx*1a while the other carried *stx*2a. Both were negative for any of the attaching genes (*eae*, or *saa* genes), and therefore considered low risk for causing infection in a healthy individual. This demonstrates that a single characteristic (e.g. ST or serotype) is not enough to make an inference of the potential pathogenic trait of any STECs (http://www.fao.org/documents/card/en/c/CA0032EN). The better way is to take all the information into consideration (ST, *stx* type, attaching genes, serotype, etc) in order to make a more informed prediction of the pathogenic potential of any STEC in conjunction with historical available data on clinical cases. For example, a strain of O113:H21 *stx*2a positive that doesn’t possess *eae* but has *saa* (a gene that encodes an auto-agglutinating adhesion) and has been linked to HUS cases [14], could be potentially harmful to humans. A similar analysis could be done in the case of any STEC that has all those attributes but has not been linked to any human cases. Even though we cannot predict the actual outcome of an infection with this strain, it still warrants a warning about its presence in foods that are consumed raw as is the case with fresh produce.

We tested for 95 known virulence genes [27] found in the most common *E*. *coli* pathotypes and did not find any genes present that would characterize the strains as STEC/EAEC//ETEC/EIEC hybrids. Among the adherence factors, *eae* and *saa* genes were found in 24% and 26% of the STEC strains, respectively. Strains that carry *eae* did not carry *saa*, and vice versa, as perviously observed for STEC isolated from fresh produce [23]. Regarding the presence of Shiga toxin type, there was great variation with most strains (67%) carrying only stx type 2, 19% carrying only stx type 1 while 15% carried both stx types. Among the *stx*2 there were 144 that were either a,d, or c variants, which are the *stx2* variants found among clinical cases [31,32,52–54] and that have a specific tropism for humans [53]. The remaining 40 STECs carrying *stx* type 2 alone were *stx* variants e, d/e and g which have been found in animal reservoirs [55]. The remaining putative virulence genes were sporadically found with the most common *exhA* gene found in 61% of the STECs, while *espP* was found in 43% of the STECs. These two genes can be found in the virulence plasmid and appear to participate in STECs infection in humans [9,11,15]. In summary, 46 of the STECs analyzed in this study carried a combination of *stx2*a/d and *eae* genes. Many researchers and food safety institutions considered a STEC with this virulence gene combination to possess an elevated risk to humans [22,53]. On the other hand, 94 of these STECs carried a combination of *stx2a/d/c* and *saa* genes. In this case, the risk is harder to determine and are considered of moderate risk if serotype was previously found associated with human cases.

We also confirmed the presence of antimicrobial resistance (AMR) genes in some of the analyzed STEC strains with a low prevalence (12%). However, those few strains carried multiple antimicrobial resistance genes. The presence of strains carrying multiple AMR genes is worrisome since they can be shared amongst other *E*. *coli* and could possible participate in the dissemination of AMR in their environments, as has been observed for tetracycline genes in *E*. *coli* isolates from beef cattle [56], for colistin resistance (*mcr*-1 gene) through plasmid-mediated transfer [57], and for ampicillin resistance genes in *E*. *coli* in an infant treated with antibiotics [58].

Phylogenetic analysis by a custom cgMLST analysis of these 276 STECs confirmed the MLST *in silico* analysis, with many different defined clades among these STECs isolated from FRFDA. The cgMLST analysis is a fast method of analysis and provides an initial visualization of the relationships among the strains analyzed. Comparable results have been observed for establishing fast relationships among genomes from diverse bacterial pathogens [27,29,59–64]. A further analysis using only the genomes of strains that are located within each individual or among selected clades can produce a more detailed evolutionary history, using single nucleotide analyses. This SNP analysis can help in determining the potential source, phylogenetic nature, lineage, and timeline of transmission of each group, as has been shown for the ST36 lineage of *Vibrio parahaemolyticus* [65].

EPECs are the leading cause of infantile diarrhea in developing countries [66,67]. EPEC strains do not carry *stx* genes but typical EPECs (tEPEC) have *eae* and *bfp* genes, and their main reservoir is humans [68]. The *eae* gene is located in the chromosome, in the LEE operon, while the *bfp* operon is typically located in the large EPEC adherence factor (EAF) virulence plasmid [68]. These tEPEC also carry the *perA* gene, which increases the expression of LEE elements [68,69]. Interestingly, 11% of our presumptive STECs were shown by *in silico* analysis to be atypical EPECs (aEPEC). Among their unusual features are the absence of the EAF plasmid, and their reservoirs can be animals or humans [68]. It is possible these aEPECs might had lost their phages upon culturing, as this pattern has been observed in clinical isolates of *E*. *coli* upon sub-cultivation [70]. The aEPEC we observed in this study may have the capacity to produce A/E lesions, since they carried both the *eae* and *tir* gene, which are the effector and receptor necessary for the formation of the A/E lesion [71].

Our results suggest that finding aEPECs in food could be of particular concern, as these strains have the potential for acquiring the *stx* phage, as observed in the *E*. *coli* O104:H4 strain found in Germany [72]. That strain was an entero-aggregative *E*. *coli* (EAEC) that had acquired an *stx*2a phage, and human illnesses that resulted during 2011 became the largest known HUS outbreak of STEC-related illness in the world [72]. Similarly, an O26:H11 strain 21765, isolated in 2005 during a milk cheese outbreak in France [73] was shown to be an EPEC strain that had probably acquired a *stx*2a phage [27]. In Gonzalez-Escalona et al (2016), the authors demonstrated that some strains of *E*. *coli* O26:H11 isolated from US cattle were phylogenetically more closely related to ST29 O26:H11 EHECs but t because these did not carry the *stx* phage, they would have been classified as EHEC-like by previous methods [74]. Over the last five years, the analyses of thousands of *E*. *coli* genomes have revealed that so-called *E*. *coli* “hybrid strains”–strains that belong to one pathotype but acquire virulence markers, such as *stx* genes, from another pathotype–could be more common than previously believed.

We are heading to a new phase in surveillance of STECs in the US by using a genomic monitoring approach and the genomes of the STEC isolated from FRFDA provides a solid foundation to build upon [75] (https://www.cdc.gov/pulsenet/pathogens/wgs.html). A database already exists that achieves the first goal of source tracking by using core genome information (NCBI pathogen detection tool). However, there is a need for improved databases that allow for fast analysis of the WGS data for detecting virulence genes, phages and plasmids content, as well as antimicrobial resistance genes.

In conclusion, STECs were isolated from diverse FRFDA food sources during the period study. The contamination frequency was relatively low (median 30 STEC strains isolated per year). However, fifty percent of the STECs analyzed in this study carried either a combination of *eae* plus *stx*, or *saa* plus *stx*, therefore being potentially pathogenic to humans. Moreover, those STECs carried most of the virulence genes described for STECs causing infections with a diverse range from HC (e.g. ST655 O111:H19 strains) to HUS (e.g. ST21 O26:H11 strains) [20,42]. Some others have not been described as causing disease in humans but have the potential to do so (e.g. ST342 O5:H-unknown strains) since they carried all virulence genes described in pathogenic strains (*stx1a*, *eae*-beta1, *exhA*, *tir*, and many of the T3SS effectors and non-LEE effectors) (Table 4). Nonetheless, the determination of the presence of STECs in FRFDA with the potential to cause disease in humans reinforces the need to continue surveillance of this important pathogen which is of importance for food safety and public health. Furthermore, the availability of these genomes could provide early warnings of food contamination from cattle or other animals, since some of the STEC isolated were carrying *stx2*e that have been usually observed causing edema in pigs [76] and are considered as probably non-pathogenic to humans [53]. Here we showed that WGS enabled comparisons across isolates to establish phylogeny, helped in identification of antibiotic resistance by monitoring the presence of antimicrobial resistance genes, and determined the presence of known virulence genes that have been linked with illnesses. A freely accessible dataset of high-quality reference genome sequences of FRFDA was previously unavailable. Future food safety investigations will benefit from the comparisons made possible by this WGS dataset as it allows for the monitoring of the recurrence and emergence of strains in the food supply. It is our goal to help develop a database that will allow for fast source tracking and accurate categorization (low risk or high risk) of STEC food isolates in a more comprehensive manner.

## Supporting information

S1 Table Metadata of all *E*. *coli* isolates analyzed in this study. (XLSX)

S2 Table *E*. *coli* virulence genes tested by in silico virulence typing. (XLSX)

S3 Table *in silico* characterization of STECs for presence of virulence genes and serotype. (XLSX)

S4 Table ST, serotype and virulence profile of intimin positive non-STEC isolated from FDA regulated foods (2003–2017). (XLSX)

S1 Fig Minimum spanning tree showing the relationships among all different STECs from this study. The numbers above the connected lines (not to scale) represent allele differences between strains belonging to the same ST. The isolates are colored based on different STs as labeled.
(TIF)
