# Supplementary figures and images for: Virulence gene profiles and phylogeny of Shiga toxin-positive Escherichia coli strains isolated from FDA regulated foods during 2010-2017
Source: PLoS One. 2019 Apr 1;14(4):e0214620. doi: 10.1371/journal.pone.0214620 (PMC6443163; doi:10.1371/journal.pone.0214620)

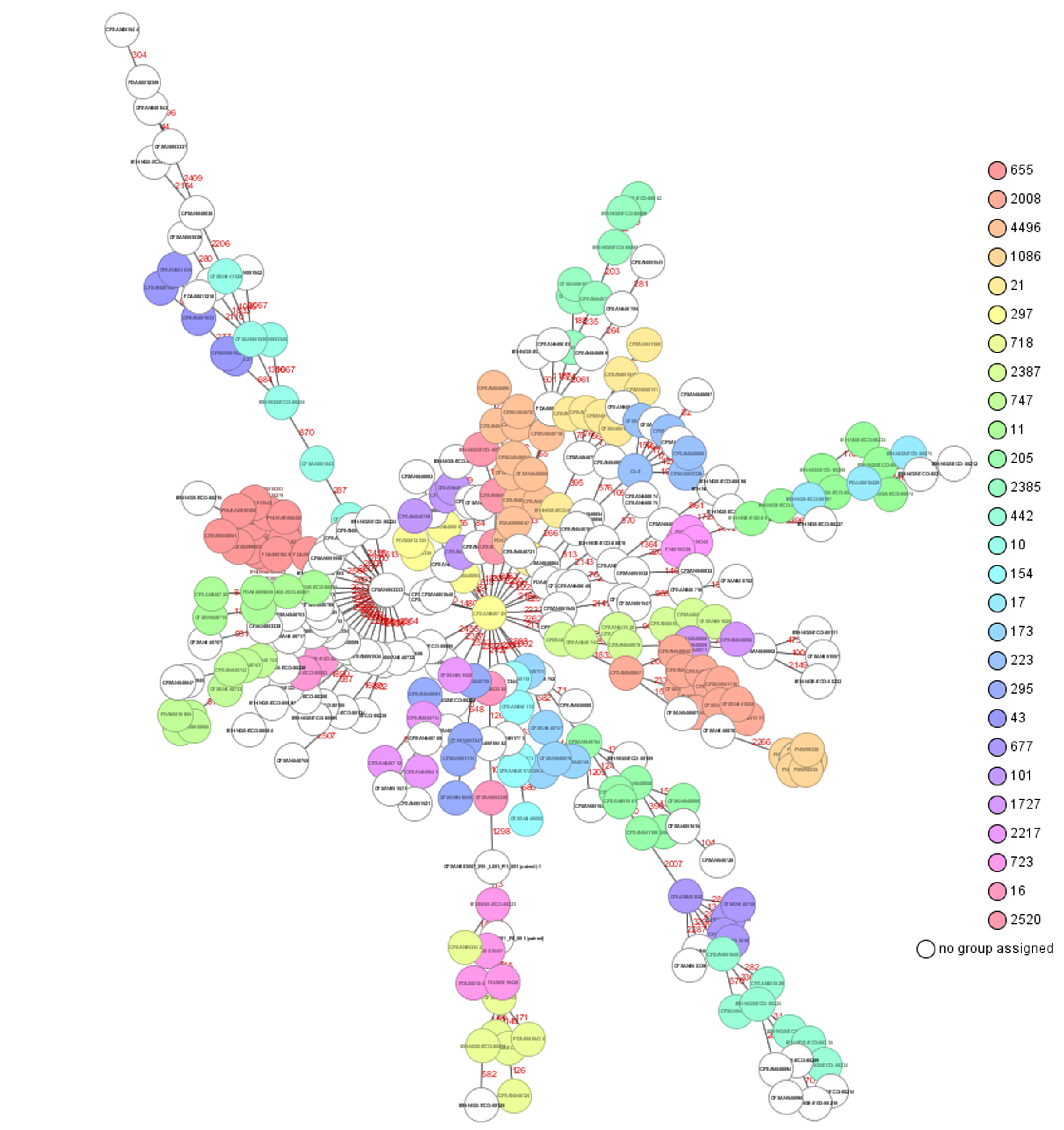

Supplement: S1 Fig — The numbers above the connected lines (not to scale) represent allele differences between strains belonging to the same ST. The isolates are colored based on different STs as labeled. (TIF) [file pone.0214620.s005.tif]
